# Amazing structure of respirasome: unveiling the secrets of cell respiration

**DOI:** 10.1007/s13238-016-0329-7

**Published:** 2016-10-14

**Authors:** Runyu Guo, Jinke Gu, Meng Wu, Maojun Yang

**Affiliations:** Ministry of Education Key Laboratory of Protein Science, Tsinghua-Peking Joint Center for Life Sciences, Beijing Advanced Innovation Center for Structural Biology, School of Life Sciences, Tsinghua University, Beijing, 100084 China

**Keywords:** respirasome structure, supercomplexes organization, cellular respiration, respiratory complexes, megacomplex

## Abstract

Respirasome, a huge molecular machine that carries out cellular respiration, has gained growing attention since its discovery, because respiration is the most indispensable biological process in almost all living creatures. The concept of respirasome has renewed our understanding of the respiratory chain organization, and most recently, the structure of respirasome solved by Yang’s group from Tsinghua University (Gu et al. Nature 237(7622):639–643, 2016) firstly presented the detailed interactions within this huge molecular machine, and provided important information for drug design and screening. However, the study of cellular respiration went through a long history. Here, we briefly showed the detoured history of respiratory chain investigation, and then described the amazing structure of respirasome.

## Introduction

Energy generation and consumption are the essential foundation of every biological process. Unlike gas or electricity used by industry, energy conversions occurred in living organisms are gentle and efficient, therefore much more complicated. According to the sources of energy flows, energy conversions can be divided into two classes. One class captures energy from light and fixes solar energy into various organic compounds, which is called photosynthesis; and the other class releases energy from organic compounds and generates the high-energy molecule ATP later consumed by virtually all other biological pathways, which is called respiration.

Photosynthesis are performed mainly in the lower grade photosynthetic bacteria, algae, and chloroplasts in higher plants (Dudkina et al., 2015). They harness solar energy and produce organic compounds containing stable chemical energy, which is the energy source of almost all animals. The processes of photosynthesis can be separated into two parts, light reaction and dark reaction. In chloroplasts, light reaction occurs on the membrane of thylakoids. Two types of protein systems, photosystem I (PSI) and photosystem II (PSII), participate in the capture of photons and generation of high-energy electrons (Dekker and Boekema 2005; Kouril et al., 2012). Those generated electrons can be transferred in two modes of electron flow: linear electron flow (LEF) and cyclic electron flow (CEF). In LEF, both PSI and PSII are involved, while only PSI participates in CEF. Both ATP and NADPH are produced by LEF, but only ATP can be produced by CEF (Johnson, 2011; Rochaix 2014). As a result, the balance between PSI and PSII can influence the final ratio of ATP/NADPH, which is important for dark reaction. Dark reaction occurs in chloroplast stroma, where CO_2_ is fixed and carbohydrates are produced (Livingston et al., 2010).

Respiration takes place in almost every living creature, and in higher organisms the executor of respiration becomes more and more sophisticated. In mammals, respiration is carried out in mitochondria. Through various catabolism pathways, carbohydrates, lipids, cholesterol, and some kinds of amino acids are catalyzed into small electron donor molecules, such as NADH and FADH, and subsequently these electron donors enter the respiratory chain located on the inner mitochondrial membrane (IMM) (Genova and Lenaz 2014). Via the respiratory chain, electrons are finally transferred to oxygen, and the energy released this way is used to pump protons from mitochondrial matrix (MM) to intermembrane space (IMS), thus forming the electrochemical gradient of proton. Ultimately, ATP synthase (complex V, CV) uses the energy within this gradient to synthesis ATP, and all these procedures together are termed as respiration (Mitchell 1961). The recognition of respiratory chain complexes and analyzing of their organization went through a long and detoured history.

Currently, it is believed that four classes of protein complexes which are relatively independent in function constitute the respiratory chain. They are complex I (NADH: ubiquinone oxidoreductase, CI), II (succinate: ubiquinone oxidoreductase, CII), III (ubiquinone: cytochrome c oxidoreductase or bc1 complex, CIII), and IV (cytochrome c oxidase, CIV). According to latest structural researches, these four classes of protein complexes are very likely to interact with each other and forming a higher-order structure, respiratory supercomplex (Vartak et al., 2013). Different combination of individual CI-CIV can produce different types of supercomplexes, and the supercomplexes that can fully perform the respiration reaction (consuming electron donors and oxygen while generating water molecules) are also termed as respirasome. (Enriquez 2016). Since respiration is irreplaceable in almost all living creatures, mutations occurred within respiratory chain complexes can lead to various severe physiological defects directly or indirectly. In the following sections, we’ll first briefly describe the research history of respiratory chain, and then analyze the structure of respirasome that are determined in our latest work.

## Recognition of respiratory chain complexes in history

### Discovery of basic respiratory chain elements in the early stage

From the early days of the 20th century, scientists successively uncovered many kinds of redox enzymes and prosthetic groups responsible for electron transfer. In 1900, Michaelis from America found mitochondria could be stained by Janus Green B. (Ernster and Schatz 1981). The staining ability of Janus Green B is dependent on the its redox state, therefore it was speculated later that mitochondria was the place where cellular respiration occurred. A couple of years later, Warburg coarsely extracted mitochondria from cavy hepatocyte for the first time in 1912, and identified several enzymes capable of conducting redox reactions, thus denominating them as respiratory enzymes (Ernster and Schatz 1981). Till the 1940s, Hogeboom firstly isolated the morphologically well-preserved mitochondria and demonstrated the location of succinoxidase and cytochrome c oxidase (Ernster and Schatz 1981). In the 1950s, the coupling sites of respiratory chain were largely recognized (Ernster and Schatz 1981). Gradually, scientists realized that cell respiration was not performed by some specific types of enzymes, but by a large series of enzymes and prosthetic groups forming the respiratory chain (Ernster and Schatz 1981).

**Figure 1:**
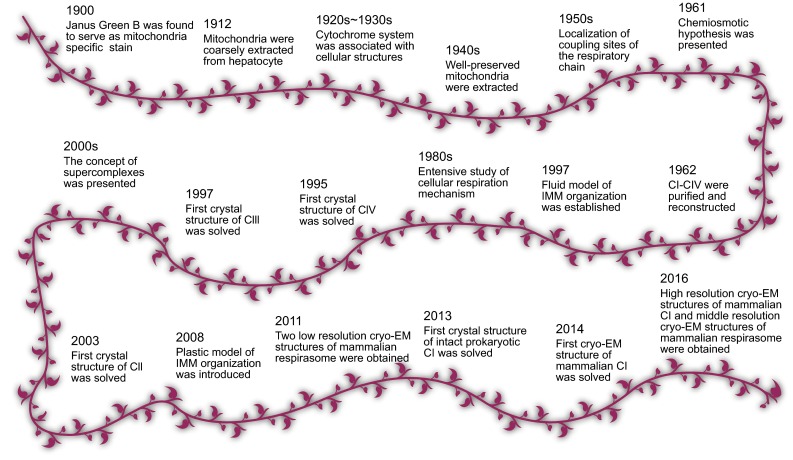
Brief research history of the respiratory chain

Till the 1960s, substantially all the prosthetic groups in the respiratory chain were identified, and their precise order in the chain was also determined through methods like measuring the standard redox potential of each element, comparing the oxidation state of each element when the reaction reaches balance, and using inhibitors aimed at specific electron acceptors. (Ernster and Schatz 1981). Instead of reacting separately, the enzymes and prosthetic groups assemble into functional modules performing energy transducing roles coordinately. Totally 4 functional modules were purified and reconstructed by Hatefi et al., till 1962, termed CI-CIV. (Hatefi et al., 1962) From that time on, work by Green, Tzagoloff and Hackenbrock in the subsequent twenty years established the fluid model of the IMM organization, (Green and Tzagoloff 1966; Hochli and Hackenbrock 1976) where all redox components are independent diffusible particles with the small electron carriers shuttling between the huge respiratory complexes I-IV, hence electron transport is considered a multicollisional, obstructed and long-range diffusional process (Hackenbrock et al., 1986) Due to lack of structural information, the mechanism of proton-pumping and electron-transfer within these complexes were largely unknown back then (Fig. 1).

### Structure determination of individual respiratory complexes from the 1990s

From 1995 to 2003, structures of CIV isolated from prokaryotic organisms and mammals were sequentially determined, and its function mechanism became more and more clear (Iwata et al., 1995; Tsukihara et al., 1995, 1996, 2003; Ostermeier et al., 1997; Kannt et al., 1998; Yoshikawa et al., 1998). CIV has three core subunits encoded by mitochondrial DNA, called SU1, SU2 and SU3. SU1 has two heme centers (heme *a* and heme *a*_*3*_) and one Cu center (CuB). Heme *a*_*3*_ and CuB together form a binuclear center, which is the site for oxygen reduction. SU2 contains a second Cu center (CuA) which comprises of two Cu atoms and accepts the electrons delivered from cytochrome c. SU3 has no redox centers but can interact with SU1. The other 11 subunits of CIV were considered to stabilize the whole enzyme. Electrons from cytochrome *c* were first transferred to CuA, then to the low spin heme a, subsequently to the high spin heme *a*_*3*_, and finally to [Fe-Cu] center where O_2_ was captured and reduced. There exist two kinds of proton pathways. Through one pathway protons were delivered to oxygen reduction center and fixed into water molecules, and by the other pathway protons were pumped from matrix to IMS directly through conformational change caused by heme *a* reaction. The first pathway is called K/D-pathway, through which protons from matrix were transferred via Lys354 or Asp124 and a series of hydrogen bonds to heme *a*_*3*_ and [Fe-Cu] center. (Tsukihara et al., 1996; Yoshikawa et al., 1998). The second pathway is called H-pathway, where His413 at the matrix side and Asp 51 at the IMS side played important roles. Before heme *a* was reduced, protons from matrix could access Asp51 via a net of hydrogen bonds and one peptide bond inside the hydrogen bonds network can prevent reverse transfer. When electrons delivered to heme *a*, the conformational change of CIV occurred, and protonated Asp51 was exposed to the IMS side, where the proton can be released through a set of hydrogen bonds (Tsukihara et al., 2003; Kaila et al., 2011). With every one electron transferred from cytochrome *c* to oxygen molecule, one proton is transferred to the [Fe-Cu] site and another proton is translocated from matrix side to IMS side. But the reason why there are two pathways (K and D pathways) responsible for oxygen reduction is not clear, and the functional difference between these two pathways is not know either (Fig. 1).

Structures of CIII have been obtained from bovine, chicken and rabbit heart since 1997, (Xia et al., 1997; Iwata et al., 1998) and till 2003 subsequent studies even got CIII crystal structures bond with either cytochrome c or inhibitors (Zhang et al., 1998; Gao et al., 2002, 2003; Lange and Hunte 2002; Palsdottir et al., 2003). Many hypotheses have been proposed to describe the mechanism of proton translocation and electron transfer within CIII, among which the Q-cycle theory has the largest influence. Every CIII has three conserved subunits with active redox centers. They are cytochrome b (SU3) containing heme *b*_*H*_ and heme *b*_*L*_, cytochrome *c*_*1*_ (SU4) containing heme *c*_*1*_, and ISP (SU5, Rieske protein, iron-sulfer cluster binding protein) containing one [2Fe-2S] cluster. These 3 subunits form the core of CIII and are responsible for the electron transfer and redox-coupled proton translocation function, with the other 8 accessory subunits holding them together. According to the Q-cycle theory, there exist two kinds of Q binding sites, including Q_o_ site, which is near the IMS side and QH_2_ is oxidized to Q by heme *c*_*1*_, and Q_i_ site, which is near the matrix side and Q is reduced to QH_2_ by heme *b*_*H*_. Three configurations of CIII were also classified, according to the distance between ISP, heme *b*_*L*_, and heme *c*_*1*_. In b position state, ISP is close to heme *b*_*L*_, while in c position state, ISP is close to heme *c*_*1*_. In the third state, int state, ISP is relatively far from both heme *b*_*L*_ and heme *c*_*1*_ (Zhang et al., 1998). When no Q or QH_2_ were bond to CIII, ISP is in the int state. After QH_2_ binded to Q_o_ site, QH_2_ is deprotonated to QH- and ISP was moved to the b position state, in the meanwhile QH^-^ delivered one electron to ISP to form semiquinone. After the semiquinone bonded to [2Fe-2S] cluster in ISP moved to the b position sate, the semiquinone delivered the second electron directly to heme *b*_*L*_, and the formed Q was released from the [2Fe-2S] cluster. Then, the reduced ISP moved to the c position sate, where the first electron rapidly transferred from the [2Fe-2S] cluster to heme *c*_*1*_, then to cytochrome *c*. The electron delivered to heme *b*_*L*_ was further transferred to heme *b*_*H*_ to reduce Q to QH_2_ at Qi site. After the electron transfer, ISP moved back to its int state, preparing to react with the next QH_2_. In one Q cycle, one QH_2_ binds to Q_o_ site, releases two protons to IMS, and delivers one electron to cytochrome c and one electron to Q at Q_i_ site, where two electrons are needed to reduce Q to QH_2_ (Iwata et al., 1998). So, in one Q cycle only one electron is delivered to one cytochrome c and one proton is consumed from matrix, but two protons are released to IMS. The reason why only one of the two electrons from QH_2_ can be delivered to cytochrome*c* is not interpreted, which seems very inefficient (Fig. 1).

The next breakthrough happened in 2003, when the crystal structure of prokaryotic version CII was solved (Yankovskaya et al., 2003). 2 years later, CII was purified from porcine and avian hearts, with the resolution of 2.4 Å and 2.1 Å respectively (Sun et al., 2005; Huang et al., 2006). Eukaryotic CII consists of a soluble heterodimer domain and an integral transmembrane region. The soluble domain contains Fp and Ip subunits, with the Fp subunit binding the FAD cofactor and the Ip subunit containing 3 Fe-S clusters. The transmembrane domain also comprises two subunits, CybL and CybS, with one heme b buried inside. To be brief, in the Fp subunit succinate is dehydrogenated to fumarate, with two electrons being extracted by FAD and transferred through three Fe-S clusters to the UQ binding site formed by IP, CybL and CybS (Sun et al., 2005). Two protons are generated at the matrix side by succinate oxidation and two other protons consumed at the IMS side by ubiquinone reduction, thus with no net proton translocated. CII is considered as a branch of the respiratory chain, and there exist many CII-like auxiliary electron donors, which participate in different metabolism pathways, including flavoprotein: ubiquinone oxidoreductase in β-oxidation, dihydroorate dehydrogenase in pyrimidine synthesis, choline dehydrogenase in glycine metabolism, sulfide:ubiquinone reductase in sulfur and seleno-amino acid metabolism, proline dehydrogenase in arginine and proline metabolism, and glycerol-3-phosphate dehydrogenase in shuttling reducing equivalents from cytoplasm (Enriquez, 2016; Lenaz and Genova 2010) (Fig. 1).

From 2006 to 2013, Sazanov’s group gradually solved the structure of the intact CI from prokaryotic cells to high resolution, (Sazanov and Hinchliffe, 2006; Baranova et al., 2007; Sazanov, 2007; Morgan and Sazanov, 2008; Berrisford and Sazanov, 2009; Efremov et al., 2010; Efremov and Sazanov, 2011; Baradaran et al., 2013) and in 2014, Hirst’s group determined the structure of bovine CI at a medium resolution (Vinothkumar et al., 2014). Recently in 2016, Hirst’s group pushed the resolution of their bovine CI structure to 4.2 Å, (Zhu et al., 2016) and Sazanov’s group got a 3.9Å resolution structure of CI from ovine heart mitochondria (Fiedorczuk et al., 2016) Moreover, via sub-region refinement, our group can also get a 3.96 Å density map of CI from porcine mitochondria (Gu et al., 2016). Mammalian CI has 45 subunits in total, among which 14 are core subunits encoded by mitochondrial DNA, and the other 31 are supernumerary subunits. All these structures show the intact structure of CI to be L-shaped, with a matrix arm and a membrane arm. The core subunits of CI form a foot-like structure slipping into a sandal-like structure composed by the circling supernumerary subunits (Gu et al., 2016). The matrix arm containing NDUFV1, NDUFV2, NDUFS1, NDUFS2, NDUFS3, NDUFS7 and NDUFS8 accommodates FMN molecule and the Fe-S clusters, which oxidizes NADH and transfers electrons to the Q reaction site. The proximal membrane subunits ND1, ND2, ND3, ND4L and ND6 catalyze the reduction of ubiquinone and couple the energy released from Q reduction to proton pumping through long-range conformational change. The distal membrane subunits ND4 and ND5 are antiporter-like proteins and perform the duty of proton pumping. ND2 is another antiporter-like protein, and together with the presumed channel formed by ND1 and ND4L, CI have four proton channels in total, but accurate proton pumping pathways are not identified yet and convincing coupling mechanism is lacking. (Fiedorczuk et al., 2016). The supernumerary subunits of CI were considered to stabilize CI and facilitate CI assembly. (Zhu et al., 2016) (Fig. 1).

## Respiratory supercomplex model

### Identification of respiratory supercomplexes

In 2000, via Blue-Native PAGE (BNPG), supercomplexes (SCs) were identified and an exciting new perspective into the organization of OXPHOS system was provided by Schägger (Schagger and Pfeiffer, 2000). BNPG is suitable for detecting molecules with large molecular weight and maintains the enzyme activity (Wittig et al., 2006). After digitonin solubilization of bovine mitochondria, the supernatant was applied to glucose-gradient ultracentrifugation. Then each layer from the centrifuge tube was used as a sample to run BNPG. Above the 1 MDa bands representing CI, many larger molecular weight bands still exist. Together with results from second dimensional SDS page and Western blot, these bands were identified to contain subunits from CI, CIII and CIV. Very soon, many groups used similar procedure to identify supercomplexes from bacteria, yeast, plant and mouse, and different patterns of supercomplex composition were reported, (Schagger and Pfeiffer, 2000, 2001 Schagger, 2001; Schagger, 2002; Pfeiffer et al., 2003; Wittig et al., 2006; Nubel et al., 2009; Zhang et al., 2005; Heinemeyer et al., 2007; Stuart, 2008; Daoud et al., 2012; Ramirez-Aguilar et al., 2011; Dudkina et al., 2006; Eubel et al., 2004; Eubel et al., 2003; Gomez et al., 2009; Acin-Perez et al., 2008; Lapuente-Brun et al., 2013; Wenz et al., 2009; Stroh et al., 2004). Technically, supercomplexes that are able to fulfill the respiration activity are also termed respirasomes.

Schägger proposed that the OXPHOS complexes are not randomly scattered in the IMM, but assembled into higher-order structures, which is called the solid model (Schagger and Pfeiffer, 2000; Chance et al., 1963). In this model, CI, CIII and CIV can be assembled into different forms of supercomplexes, including I_1_III_2_IV_1_, I_1_III_2_IV_2_, I_1_III_2_IV_4_, III_2_IV_1_, and III_2_IV_2_. Those detected free forms of respiratory complexes were interpreted as assembly intermediates (Fig. 1).

Since the definition of SC, various evidence suggesting the advantages of forming SC has been accumulated. The advantages appear in four aspects (Dudkina et al., 2015; Genova and Lenaz, 2014; Enriquez, 2016; Lenaz and Genova, 2010; Cogliati et al., 2016; Moreno-Loshuertos and Enriquez, 2016; Genova, 2014; Sazanov, 2015; Liao et al., 2015): (1) CIII and CIV integrated onto CI to form SC can stabilize CI. (2) SCs produce much less ROS (reactive oxygen species). (3) The catalytic activity of individual components is higher in SCs. (4) Through substrate channeling, the efficiency of electron transfer is elevated in SCs. In 2002, Lamantea and his colleagues discovered that some CIII abnormalities not only disrupt the activity of CIII, but also hamper the proper function of CI (Lamantea et al., 2002). In 2004, Acìn-Perèzand his colleagues reported that when CIII is ablated, CI is prone to degradation in mitochondria (Acin-Perez et al., 2004). Subsequently, other groups confirmed this phenomenon, and lack of cytochrome c was found to disrupt the assembly and stability of CI and CIV (Vempati et al., 2009). In 2012, Moreno and his colleagues found that partially assembled CI can interact with CIII and CIV, and they claimed that completion of CI assembly requires interaction with CIII and CIV (Moreno-Lastres et al., 2012; Diaz et al., 2006). The prevention of ROS formation is another advantage of CI and CIII assembling into SC. The ROS generating sites are considered to be FMN and N2 in CI and Q_o_ site in CIII, where semiquinone can be formed and oxygen is accessible. (Genova, 2014) In 2013, Maranzana and his colleagues reported that in both purified bovine mitochondria and reconstituted liposomal preparations, the disruption of SC strongly enhance the generation of superoxide and H_2_O_2_ by CI. (Maranzana et al., 2013) The structural explanation of SC preventing ROS formation is still lacking. There are also some implications that SCs can enhance the catalytic activity of their component complexes. In 2006, Schägger and his colleagues found that the activity of CI and CIII is higher in SCI_1_III_2_IV_1_ compared to the SC lacking the terminal oxidase, I_1_III_2_ (Schafer et al., 2006).

Evidence for substrate channeling in SC have been growing. Interaction between CI and CIII can cause segmentation of Q pool. Ubiquinone functions as the center of electron transfer to CIII, because CI, CII and CII-like auxiliary enzymes can all donate electrons to Q to form QH_2_, but QH_2_ can only deliver electrons to CIII. Thus, the allocation of Q pool is of vital importance in regulating different metabolism pathway (Enriquez, 2016; Moreno-Loshuertos and Enriquez, 2016). One group estimated that 15% to 30% of the total Q molecules were bound to proteins and the remaining fraction was likely to be free in membrane (Lass and Sohal, 1999; Lass et al., 1999; Lass et al., 1999). In 2008, Rossigol and his colleagues demonstrated that a portion of the total Q pool was not able to be used for succinate oxidation (CII function). They estimated that the portion is 79% in muscle mitochondria and 21% in liver mitochondria (Benard et al., 2008). In 2013, Lapuente and his colleagues found that when the amount of CIII is less than the amount of CI, oxidation of succinate and glycerol-3-phosphate is blocked but oxidation of NADH remain unchanged (Lapuente-Brun et al., 2013). These results can be explained that most CIII is bound to CI to form SC, and no free CIII is available to utilize the membrane Q pool rather than the Q pool segmented by SC. In 2014, Hirst’s group reported that the activity of CIII can be maximized only when succinate and NADH are provided simultaneously, instead of either substrate alone (Blaza et al., 2014). In 2015, Anderson and his colleagues found that the impact of shortage of ubiquinone on different substrate respiration can vary. Oxidation of glycerol-3-phosphate is mostly affected, followed by succinate, and the oxidation of NADH is the least affected (Anderson et al., 2015) All these results suggest segmentation of Q pools, but the structure basis in SC is not clear.

On the contrary, evidence for cytochrome c pool segmentation in SC between CIII and CIV are comparatively rare. The lacking of evidence for cytochrome c pool segmentation in SC may be due to the fact that most CIV are in the free form (Genova, 2014). Even if there is actually a cyt.c pool segmentation in SC, the portion will be very small and not easy to detect. But according to our latest structure of respirasome, we provided a model showing that the binding site of Cyt.*c* in CIII and CIV are located near each other within 10 nm in the IMS plane formed by TMH ends of CI, CIII and CIV, and this arrangement can provide the possible structural basis for Cyt.*c* channeling (Gu et al., 2016).

### The organization of the respiratory chain

Two models describing how the respiratory chain is organized are presented above, the fluid model (Hochli and Hackenbrock, 1976; Chazotte and Hackenbrock, 1991; Hackenbrock et al., 1984; Gupte et al., 1984; Schneider et al., 1980; Schneider et al., 1982; Schneider et al., 1982; Hackenbrock et al., 1980; Hochli and Hackenbrock 1979) by Hackenbrock and the solid model (Schagger and Pfeiffer, 2000; Schagger, 2001; Schagger and Pfeiffer, 2001; Wittig et al., 2006; Chance, 1950; Keilin and Hartree, 1947) by Schägger. Both of these models have their reasonable parts and some other parts that are not in coordination with existing experiment results. Integrating the advantages of the two models above, Antonio and his colleagues proposed the plastic model (Acin-Perez et al., 2008; Lapuente-Brun et al., 2013; Moreno-Loshuertos and Enriquez, 2016; Enriquez and Lenaz, 2014; Acin-Perez et al., 2008; Cogliati et al., 2013). In his model, the organization of OXPHOS complexes is very flexible. Both the assembled SCs and free individual complexes can perform their function, with SCs being more efficient in energy generation and less active in ROS production. The degree of free complexes integrating into SCs is very likely under elaborate regulation, to accommodate to different demands of the cell environment (Sun et al, 2016; Wang et al., 2016). Different forms of SCs may participate in different metabolic pathways. SCI_1_III_2_IV_1_ can only oxidize NADH, which is the main product of glucose metabolism, while SCIII_2_IV_1_ can receive QH_2_ from CII, which can oxidize succinate (Genova and Lenaz, 2014).

Recent studies indeed provided evidence that SC distribution is correlated with cell conditions (Vartak et al., 2013; Lapuente-Brun et al., 2013; Ikeda et al., 2013). SCAF1, previously called Cox7RP, is the first assembly factor functions only in SC assembly and not required in assembly of individual complexes. SCAF1 is required for superassembly of CIII and CIV, and in mice mutants lacking active SCAF1 no SCs requiring direct interaction between CIII and CIV exist, with almost all CIVs remain single complex form, but in these mutants no major biological problems were caused, which suggests the SCs and free complexes both function normally with the mutants probably lacking some fine regulations of energy supplication.

Moreover, experiment evidence has been given that the shape and curvature of inner mitochondria membrane, which are very dynamic, can have significant influence on SC distribution (Cogliati et al., 2013; Liesa and Shirihai, 2013). Besides, in different tissues, different species, different environmental conditions and different ages, the distribution of SC can vary significantly (Ramirez-Aguilar et al., 2011; Gomez et al., 2009; Lapuente-Brun et al., 2013; Hofmann et al., 2012; Frenzel et al., 2010; Feng et al., 2016). These heterogeneity of SC distribution is in line with the plastic model, although the correlation between specific SC distribution types and specific cell conditions is not defined yet (Enriquez, 2016).

### The structure of respirasome

Structural study of respiratory supercomplexes could be traced back to the year of 2005, when Dudkina firstly determined the structure of SCI_1_III_2_ purified from *Arabidopsis thaliana* at a resolution of 18 Å through the single-particle cryo-EM technology, (Dudkina et al., 2005) which has been proved very useful since all the later structures of supercomplexes are determined this way. The SCI_1_III_2_ they purified proved to be very stable, and when compared with the map of individual complexes from mammals, CIII in the *Arabidopsis thaliana* SCI_1_III_2_ is somehow smaller, while CI has extra knob-like protein densities attached to either side of the hydrophobic arm, which is a unique feature not seen in CI from any other species, and these extra proteins are required for the formation of supercomplex, indicating their interactions with CIII. But due to the limited resolution, Dudkina were not able to recognize the cavity responsible for ubiquinone-channeling.

Then in 2007, Jesco provided a structural model of SCIII_2_IV_1-2_ from yeast mitochondria at a resolution of 15 Å (Heinemeyer et al., 2007). In yeast where CI is lacking, CIII and CIV can form very stable supercomplexes. In their structures, CIII dimer either binds to one CIV or two CIV at different sides, the total structure showing a two-fold symmetry. The III_2_IV_2_ structure is very likely to be the brick of respiratory string. They also found cytochrome *c* bound to their structure, and they estimated that the distance between Cyt.*c* binding sites in CIII and CIV was less than 40 Å, which was different from that in mammals.

Several years later, two groups independently reported the structure of SCI_1_III_2_IV_1_ from bovine heart at the resolution of 22 Å (Dudkina et al., 2011) and 19 Å (Althoff et al., 2011) respectively in 2011. Despite the 36 Å structure of bovine SCI_1_III_2_IV_1_ solved by Schäfer in 2007, (Schafer et al., 2006) through which the location of CI, CIII and CIV could not be precisely determined because of the low resolution, these are the first structures of supercomplex from mammals, through which the arrangement of individual complexes could be easily affirmed. Both these groups used X-ray structures of individual complexes to build the model, and they all calculated the distance of Q binding sites between CI and CIII as 13 nm, the distance of Cyt.*c* binding sites between CIII and CIV as 10 nm. But due to the limited resolution, detailed information about interactions among CI, CIII and CIV could not be extracted (Fig. 1).

**Figure 2:**
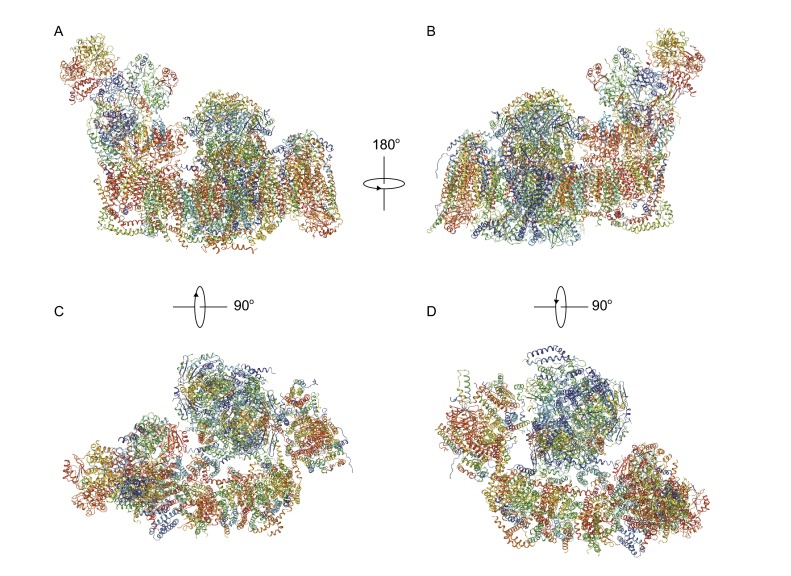
Overall structure of respirasome. The structure of respirasome from porcine heart at a resolution of 5.4 Å. (A) front view; (B) back view; (C) top view; (D) bottom view. The relations between the views are indicated by the angles

Recently, our lab solved the structure of SCI_1_III_2_IV_1_ (respirasome) from porcine heart at an overall resolution of 5.4 Å, with the resolution of both individual CI and CIII within the respirasome reaching 3.96 Å using sub-region refinement (Gu et al., 2016). Aided by the 3.96 Å density map of CI, we were able to accurately assign all the 14 core subunits together with 20 supernumerary subunits, and build 17 additional backbone models into the unoccupied density. Totally, 77 TMHs from CI were identified. Our model of CI was proved to be correct by the latest work performed by Zhu and Fiedorczuk (Zhu et al., 2016; Fiedorczuk et al., 2016). In the 3.96 Å density map of CIII, the distances between [2Fe-2S] and heme*c*_*1*_, [2Fe-2S] and heme*b*_*L*_ are 30 Å and 27 Å respectively, which suggests the CIII in our structure is in the ‘int’ state. Unfortunately, no density for Cyt.*c* was found in our map (Fig. 2)

.

When compared with CI in the free form, the matrix arm of CI and the distal end of CI’s membrane arm bend a little towards the central part of CI’s membrane arm in the respirasome, to better interact with CIII and CIV. CIII form a dimer in the respirasome, located at concave side of CI’s membrane arm. The CIII dimer has a two-fold axis, which is perpendicular to the membrane plane, with the matrix domains of CIII protruding around 75 Å out of the membrane and facing the matrix arm of CI. CIV sits at the distal end of CI’s membrane arm, and likely due to its flexibility, its density map is of relatively low resolution. Altogether, 77 TMHs from CI, 26 TMHs from CIII and 28 TMHs from CIV were identified, and the TMHs of the three complexes form an enormous transmembrane disk with their ends at the intermembrane side aligned to form a plane (Gu et al., 2016).

The interaction between CI and CIII is rather stable. Within our structure, we can affirm that NDUFA11 of CI directly interact with both CI and CIII. The central part of NDUFA11 contains 4 TMHs, with an extra short helix at the N-terminus and a loop region at the C-terminus. The central TMHs of NDUFA11 bundle together and parallel the TMH domain of CI’s membrane arm. At one side, the N-terminus of NDUFA11 interacts with ND5, the C-terminal loop contacts ND2, and TMH4 interacts with ND4. At the other side, NDUFA11 is close to UQCRB and UQCRQ of one CIII in the dimer. These features agree with previous reports that a mutation disrupting TMH1 of NDUFA11 destabilized the entire complex, and that blockof NDUFA11 expression disrupted the assembly of CI, resultingin the accumulation of 550 kDa and 815 kDa CI sub-complexes. At another site, the N-lobe of NDUFB9 glues CI and CIII together. A short loop of UQCRC1 (subunit of CIII) inserts into the groove circled by the N-lobe of NDUFB9 and an unassigned subunit of CI, while the LYR-motif in the N-lobe of NDUFB9 is important for NDUFAB1 (subunit of CI) binding. These features are in accordance with previous data that mutations in NDUFB9 can lead to CI deficiency, NAD^+^/NADH imbalance and tumor metastasis. Gene blast shows the binding motifs in NDUFA11, NDUFB9 and UQCRC1 are highly conserved across species, suggesting their important roles in supercomplex formation (Gu et al., 2016).

CIV binds relatively loosely to CI and CIII. The TMH of COX7C (subunit of CIV) is packed against the last TMH of ND5 at the distal end of CI’s membrane arm. Additionally, at the matrix side, the flexible region of COX7A (subunit of CIV) contact with both UQCRC1 and UQCR11 (subunits of CIII). Perhaps due to the lack of supernumerary subunits from CI functioning as bridge, these interactions are slightly weaker compared to CI-CIII interaction. Furthermore, there are clear gaps between these three complexes. According to multiple previous reports, a large amount of different lipid molecules, including cardiolipin, phosphotidylcholine, and phosphotidylethanolamine, should be present in the isolated samples, we propose these lipid molecules may occupy these gaps to further stabilize the respirasome (Gu et al., 2016).

Our structure provides evidence for substrate channeling. Ubiquinone is supposed to be trapped in the gap between CI and CIII, which is filled with lipid molecules, and this compartmental arrangement can greatly increase the efficiency of ubiquinone transport. At the IMS side, the binding site of Cyt.*c* in CIII and CIV are located near each other within 10 nm in the plane formed by TMH ends of CI, CIII and CIV. This feature vastly facilitates Cyt.*c* communicating between CIII and CIV (Gu et al., 2016) (Fig. 2).

### Evidence for respiratory strings or megacomplexes

Cryo-electron tomography and freeze-fracture EM studies in earlier years indicate the respiratory complexes could form higher order structures which might even influence the shape of mitochondria cristae (Allen et al., 1989) Richard in 1989 reported that in his fracture photos, ATPase could be seen clearly form dimers, with some other large particles in accordance with complex I (now considered the supercomplexes) regularly arrayed in rows, and many other authors showed similar pictures (Heinemeyer et al., 2007; Nicastro et al., 2000; Strauss et al., 2008; Sousa et al., 2013). The ATPase dimers in these electron microscopy photographs were somehow far more obvious than the respiratory rows, and that’s the reason why investigations into megacomplexes proved to be difficult. Years later, Schägger measured the size of individual CI, CIII, CIV, and the distance between particles in the presumed respiratory row, titrated the ratio of CI:CIII:CIV in supercomplexes, and then proposed the concept of respiratory string, where supercomplex I_1_III_2_IV_4_ is the brick, forming the dimer first and then a string linked by CIV (Wittig et al., 2006). After that, we and many others identified higher molecular weight bands than the bands of SCI_1_III_2_IV_1_ in BNPG, and classified particles from Cryo-EM images similar to the presumed respiratory dimer, or megacomplex (Gu et al., 2016; Heinemeyer et al., 2007; Sousa et al., 2013; Bultema et al., 2009; Davies et al., 2011). These results were obtained from different organisms, including porcine, potato, yeast and bacteria, suggesting the existence of a higher order arrangement of respiratory chain elements across species. Due to its large scale and highly compacted organization, respiratory string could be responsible for stabilizing the cristae structure and function very efficiently in urgent conditions (Riva et al., 2003, 2006; Perkins et al., 1997; Muhleip et al., 2016; Stroud and Ryan, 2013; Barcena et al., 2010; Chen et al., 2015).

## Conclusions and perspectives

Researches focusing on the energy metabolism have always been of great importance and interest, and after a century’s effort, scientists have got exciting achievements in understanding the respiratory chain. We know that the respiratory chain elements are arranged in a highly ordered manner, and have reached some reasonable speculations about how these gigantic protein machines work properly at an atomic level, but some critical information is still lacking. For example, the organization of respiratory chain can vary to adapt to different cell conditions, but the accurate regulatory mechanism is far from clear; in addition, accumulating evidence is indicating the existence of megacomplexes, which we know very little about besides assuming it could influence the shape of mitochondria and might function in urgent situations; moreover, in order to provide valuable information for clinical application, the structure of respirasome from human, rather than bovine or porcine is required. Massive efforts are still needed in understanding the details of cellular respiration and developing remedy plans for diseases relating to mitochondria abnormality. We believe a more delicate picture depicting the detailed information of respirasome is on its way.
